# Monkeypox: From A Neglected Tropical Disease to a Public Health Threat

**DOI:** 10.3390/idr14050079

**Published:** 2022-09-30

**Authors:** Francisco Antunes, Rita Cordeiro, Ana Virgolino

**Affiliations:** 1 Instituto de Saúde Ambiental, Faculdade de Medicina da Universidade de Lisbon, 1649-028 Lisbon, Portugal; 2 Laboratório Associado TERRA, Faculdade de Medicina da Universidade de Lisbon, 1649-028 Lisbon, Portugal; 3 Emergency Response and Biopreparedness Unit, Department of Infectious Diseases, National Institute of Health Doutor Ricardo Jorge (INSA), 1600-609 Lisbon, Portugal

**Keywords:** zoonosis, monkeypox virus, emerging infectious diseases, West and Central Africa, 2022 outbreak

## Abstract

Since the first case of human monkeypox was diagnosed in 1970, the disease remained endemic in several countries in West and Central Africa. In 1996, there was a sudden increase in cases in the Democratic Republic of Congo (DRC), and since 2017 an ongoing outbreak in Nigeria took place, probably related to the population growth, human invasion of MPXV animal habitat reservoirs, and the waning of the cross-protection offered from smallpox immunization, later ending in 1980. Since May 2022, an unprecedented outbreak of human monkeypox has rapidly spread around the world, outside endemic regions of Africa, through new modes of transmission, showing differences in clinical features compared with previous reports. The 2022 MPXV strain belongs to the clade of West Africa but diverges from the original strain, making the virus more transmissible. The authors review the main milestones in more than 50 years of history of human monkeypox, from a rare viral zoonotic infection to a public health emergency.

## 1. Introduction

In July 2022, the multi-country outbreak of monkeypox was determined by the Director-General of the World Health Organization (WHO) as a Public Health Emergency of International Concern (PHEIC) [1]. A PHEIC should be declared under three conditions: (i) if a disease outbreak is an extraordinary event, (ii) when it constitutes a public health risk to other states through the international spread, and (iii) when a coordinated international response is potentially required. Monkeypox has been circulating in several African countries for decades but only after it spread to Europe and North America in 2022 has received international attention. Table 1 presents the most relevant milestones in the history of monkeypox since 1958.

## 2. Monkeypox: A Neglected Tropical Disease

Monkeypox is a disease caused by a virus that was first discovered during a nonfatal outbreak at an animal facility in Copenhagen, Denmark, in 1958 [2]. The outbreak occurred in *Macaca fascicularis,* which had arrived from Singapore. The outbreak’s manifestations were vesiculopustular skin eruptions in the trunk, tail, face, limbs, palms of the hands, and soles of the feet. After that, several outbreaks of monkeypox virus (MPXV) were reported in a colony of captive monkeys in Philadelphia, United States of America (USA) [3], in two monkeys after exposure to whole-body irradiation [4], and a broad-host-range of monkeypox virus (MPXV) at Rotterdam Zoo, in the Netherlands [5]. Later on, an outbreak has occurred in prairie dogs, in the USA, living in close contact with various rodent species imported from Ghana, West Africa [6]. As a result, the prairie dogs transmitted MPXV to approximately 40 humans. This was the first known outbreak of human monkeypox outside of Africa.

Since its discovery, MPXV has revealed a propensity to infect and induce disease in a large number of animals within the *Mammalia* class from pan-geographical locations. So far, over 40 species have been documented to have been naturally or experimentally infected with MPXV (Table 2).

**Figure 1:**
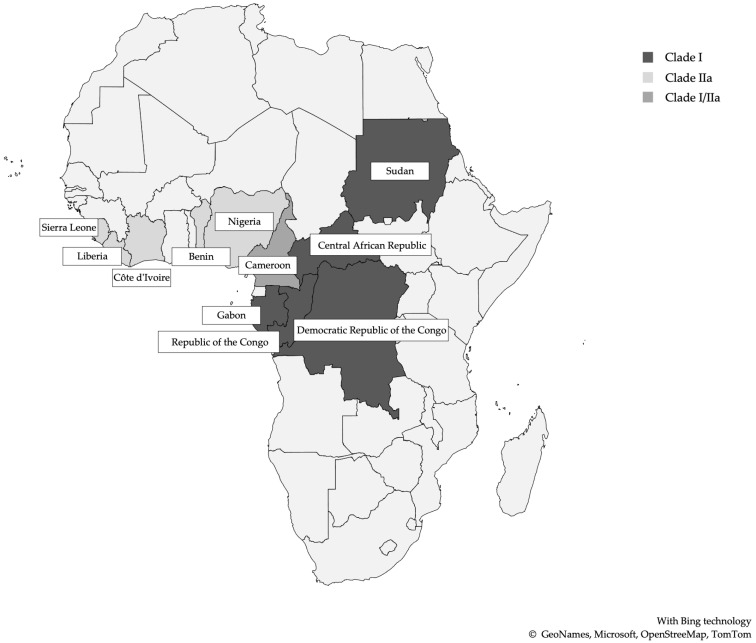
African countries with reported Monkeypox cases and clades (1970–2022) (adapted from [14]).

These orthopoxviruses, similar to the variola major virus (VARV), the causative agent of smallpox, experimentally can infect several animal species (*M. fasciculans* and *Macaca mulatta* monkeys, prairie dogs, squirrels, rabbits, and other small animals) via a variety of multiple different inoculation routes [8]. This finding has impeded the elucidations of the natural host, although the strongest candidates are African squirrels and/or other rodents. In nature, the major environs of MPXV are restricted to the Congo Basin and West Africa. Humans and highly susceptible non-human primates infected with MPXV have identical clinical manifestations compared to humans infected with the smallpox virus. Historically, MPXV genetic diversity has been classified into two clades: West African and Central African clades [9]. However, a new proposal for MPXV classification has been established into three clades (I, IIa, and IIb), each with distinct geographical, clinical, genomic, and epidemiological differences and correlate with the different epidemiological MPXV outbreaks (Figure 1). Clade I corresponds to the prior “Congo Basin clade”, while clades IIa and IIb correspond to the “West Africa clade” [10,11] (Table 3). The clades I and IIa include the majority of monkeypox virus linked to outbreaks from Central and Western African outbreaks and localized spillover events in global north countries from both human and non-human hosts [10]. Clade I is more clinically severe, with higher mortality rates, and exhibited increased transmissibility. Conversely, Clade IIa MPXV genomes are associated with milder infection, lower mortality rates, and reduced transmissibility [9,12,13]. Clade IIb includes isolates originating from the 2017–2019 outbreaks and genomes from the most recent 2022 outbreak, diverging emerging lineages that are currently under investigation [10,12].

Campaigns for smallpox eradication during the 1960s and 1970s in the Democratic Republic of Congo (DRC) (ex-Zaire) were successful, eradicating the disease in 1971 [15]. Nearly two years after the last case of smallpox on September 1st, 1970, a 9-month-old child suspected of having smallpox was admitted to Basankusu Hospital, Equatorial Province, DRC [16]. According to records, skin lesions were hemorrhagic, showing a centrifugal distribution typical of smallpox. During the scabbing stage, the child developed enlarged, painful cervical nodes. The rash lasted about two weeks, and specimens from the patient were sent to the WHO Smallpox Reference Center in Moscow, Russia, where an MPXV was isolated. This was the first recognized case of human infection caused by MPXV. Basankusu Town is located in a dense tropical rain forest, and monkeys are eaten from time to time and considered a great delicacy in Bokenda Village, where the youngest child and the family lived. Since then, monkeypox has become endemic to DRC and has spread to other African Countries, mainly in West and Central Africa. Before 1970, monkeypox was recognized only in non-human hosts.

From 1970 to 1979, 47 human monkeypox cases were reported in West and Central Africa, of which 38 were referred from DRC, all occurring in the tropical rainforest and associated with animal contact [17]. Moreover, only 9% of the patients had a smallpox vaccination scar, having been vaccinated more than five years before. Twenty-three (49%) had severe disease, and eight (17%) of the patients died from monkeypox during the acute illness. After the declaration of smallpox eradication, in 1980, by the World Health Assembly, WHO sponsored enhanced human monkeypox surveillance efforts in the central regions of DRC between 1981 and 1986, during which 138 cases were identified, and the overall mortality rate was high as 9,8% in children not vaccinated against smallpox. Seventy-two percent of the cases were zoonotic transmissions, and most of the cases occurred in children with an average age of 4.4 years [18]. Since the end of the WHO monitoring project in 1986, reports of the persistent occurrence of monkeypox in humans have decreased. However, in 1996, there was a sudden increase in the number of human MPXV-infected cases reported in DRC [19,20]. Between 2006 and 2007, in DRC, MPXV transmission increased 20-fold since the 1980s, smallpox-vaccinated people showed a 21-fold lower risk of infection than unvaccinated people, and zoonotic transmission occurred in most cases [21]. Remarkably, another outbreak started in Nigeria in 2017, signaling the re-emergence of the West African clade of human monkeypox in the country, after 38 years since it was last reported. This outbreak was characterized by predominant infection among young adult males (69%) and significant person-to-person secondary transmission [22]. On the other hand, a substantial number of cases were young adults of reproductive age presenting genital ulcers, as well as concomitant syphilis and HIV infection. It was not excluded from sexual transmission, in some cases, through close skin contact during sexual intercourse or by transmission via genital secretions [23].

At the date of November 2019, the largest recorded outbreak in West Africa was ongoing in Nigeria since September 2017 [24,25,26]. The substantial resurgence of monkeypox in Nigeria in 2017 appears to have been driven by a combination of population growth, accumulation of unvaccinated smallpox population, and a decline in smallpox vaccine immunity [25]. The outbreak likely resulted from a complex intersection of events and, given the zoonotic nature of the disease, required a robust outbreak response collaboration among humans, animals, and environmental health institutions [27]. The data available indicate that the current outbreak is either a multisource outbreak or one stemming from the previously undetected endemic transmission because the cases were not epidemiologically linked [27,28,29].

Linked with Nigeria, imported cases of human monkeypox were reported in the United Kingdom (UK), Israel, and Singapore [30,31,32]. The availability and speed of international transportation combined with the natural progression of the disease (long incubation and prodromal periods, up to 21 days combined) can increase the risk of monkeypox spreading from rural regions into urban areas and to countries outside Africa [31].

Contact with the animal reservoirs, including live or dead animals, often through the hunting and preparation of bushmeat as food, is a presumed driver of monkeypox human infection. Closer contact between humans and animals through deforestation, demographic changes, climate change, hunting, and population movement might account for the recent increase in reported cases and expansion of the geographic range. Civil war and population displacement can force inhabitants to seek alternative sources of protein, including the consumption of monkeys, squirrels, and other rodents [28].

Primary animal-to-human infection is assumed to occur when handling monkeypox-infected animals through direct (touch, bite, or scratch) or indirect contact, although the exact mechanism(s) remains to be defined [33]. The virus is assumed to enter the body through broken skin, respiratory tract, or mucous membranes (eyes, nose, or mouth). Secondary human-to-human transmission occurs presumably through large respiratory droplets or direct or indirect contact with body fluids, lesion material, and contaminated surfaces or other material, such as clothing or linens. Prolonged contact with patients renders hospital staff and family members at greater risk of infection. Nosocomial transmission has also been described [30]. As with all zoonotic diseases, a comprehensive One Health approach is necessary for disease detection, including wildlife surveillance and investigations into the animal reservoir/reservoirs.

From the point of view of clinical features, monkeypox is a similar disease presentation to smallpox in humans, with the additional distinction of lymphadenopathy. After an initial febrile prodrome, a centrifugally distributed maculopapular rash develops, with lesions often present on the palms of the hands and soles of the feet. The infection can last up to four weeks until crusts separate and a fresh layer of the skin is formed. The rash undergoes several stages of evolution, from macules, papules, vesicles, and pustules. The primary differential diagnosis is severe chickenpox with lesions in the palms and soles [34]. The lesions in chickenpox are more superficial and occur in clusters of the same stage, with denser manifestations on the trunk than on the face and extremities. A clinical sign differentiating monkeypox from smallpox and chickenpox is the presence of large lymph nodes, particularly cervical, axillar, and inguinal nodes [35] (Table 4). Severe cases occur more commonly among children and are related to the extent of virus exposure, patient health status, and severity of the complications. Complications include secondary bacterial infections, respiratory distress, bronchopneumonia, gastrointestinal involvement, dehydration, encephalitis, and ocular infection, which can result in permanent corneal scarring [28].

An early differential and rapid diagnosis is important to recognize and restrict the spread of the virus in the community. Furthermore, it is pivotal to control the outbreak and the epidemic of MPXV because clinical manifestations of MPXV infection are difficult to distinguish from the other poxvirus-caused diseases.

Testing for the presence of MPXV should be performed in appropriately equipped laboratories with staff trained in relevant technical and safety procedures [36]. All manipulations in laboratory settings of specimens originating from suspected, probable, or confirmed cases of monkeypox should be conducted according to a risk-based approach [37]. Specimens should be packaged and shipped to these laboratories following national and international requirements [38].

Suitable samples for diagnostic testing are skin lesion material, including swabs of lesion exudate, roofs from more than one lesion, or lesion crusts. In addition to a lesion specimen, the collection of an oropharyngeal swab is recommended. Other clinical samples can be considered for research purposes, such as urine, semen, rectal and/or genital swabs, on indication based on the clinical presentation, including the location of lesions and EDTA blood in the prodromal period and before skin lesions become manifest. Two serum or plasma samples could also be collected, at least 21 days apart, with the first being collected during the first week of illness [36].

In the laboratory, MPXV can be diagnosed by using techniques such as nucleic acid amplification testing (NAAT), viral culture/isolation, electron microscopy, serological analysis for specific antibodies (immunoglobulin G [IgG] and Immunoglobulin M [IgM] based), and immunohistochemistry (antigen detection) (Table 5).

Among the diagnostic tests, NAAT is the preferred laboratory test, given its accuracy and sensitivity. Currently, a variety of protocols have been developed for NAAT, such as real-time or conventional polymerase chain reaction (PCR). NAAT can be generic to Orthopoxvirus (OPXV) or specific to MPXV [36]. As an additional molecular tool, whole-genome sequencing should be used to reconstruct the genome sequences and to inform about the evolutionary trajectory of the monkeypox outbreak using phylogenomic data, being useful for epidemiological purposes [12].

Viral isolation, electron microscopy, and immunohistochemistry are techniques that require advanced technical skills and training and should be reserved to reference laboratories with high containment facilities and appropriate expertise and skills [39].

Serological testing is also useful for the epidemiological purpose providing a broad window of detection relative to direct virus detection, although it is limited by cross-reactivity to a variety of OPXV. Anti-OPXV IgG antibodies detection does not allow a definitive diagnosis since a previous exposure to OPXV or smallpox vaccine could cause a false positive result. In the case of recent exposure to MPXV, the detection of anti-OPXV IgM could be helpful, also in individuals with prior vaccination, but it is also limited by the absence of OPXV specificity [40].

## 3. Monkeypox: A Public Health Threat

Up to May 2022, monkeypox outbreaks have occurred in several countries around the world, leading to a strong vigilance of scientists [41]. All the four episodes of human monkeypox outside endemic areas were related to the West African clade; one, the outbreak in the USA (2003), associated with rodents infected imported from Ghana, and the other three with the ongoing outbreak in Nigeria, starting on September of 2017.

The 2022 monkeypox outbreak in Europe and North America is part of the outbreak of human monkeypox caused by the West Africa clade of the MPXV. The outbreak reached Europe on 6 May 2022, when the UK reported its first case of monkeypox, a person who traveled to Nigeria before a diagnosis. However, many of the new confirmed cases had no history of travel to Nigeria or Africa, suggesting the initiation of a community transmission [42]. This was probably the first case of human monkeypox related to the ongoing outbreak in Europe and North America. Globally, as of September 2022, the total number of human monkeypox cases was 31,800; of these, 31,425 occurred in countries that have not historically reported cases. The three leading countries that have reported cases of this outbreak (representing nearly half of the total reported cases) are the USA, Spain, and Brazil [43] (Table 6). The total number of confirmed deaths was 20, and, of these, eleven were in locations that have not historically reported monkeypox.

The unexpected appearance of monkeypox and the wide geographic spread of cases indicate that the MPXV might have been circulating in levels below the detectable threshold by the surveillance systems, and that sustained human-to-human transmission might have been undetected for some time [44]. Phylogenetic analyses suggest that the virus circulated undetected for some time outside areas where it has been endemic, possibly masquerading as other sexually transmitted infections (STIs) [45].

Routes of MPXV transmission include human-to-human via direct contact with infectious skin or mucocutaneous lesions, respiratory droplets (and possibly short-range aerosols), or indirect contact (also described as fomite transmission) from contaminated objects or materials. While it is known that close physical contact can lead to transmission, there is no clear evidence of sexual transmission through seminal or vaginal fluids [44]. However, vertical transmission (mother-to-child) and fetal deaths have already been described [46].

**Figure 2:**
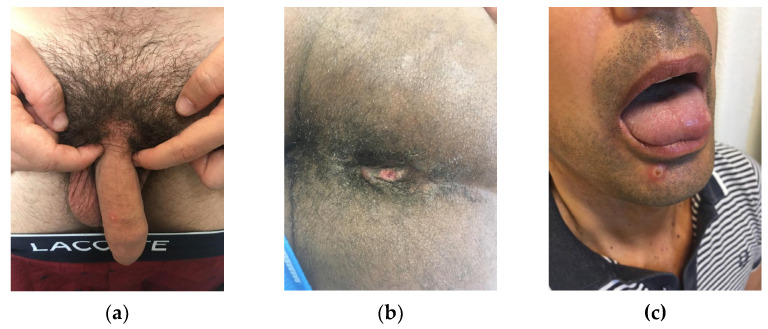
Genital (**a**), anal (**b**), and mucocutaneous (**c**) monkeypox lesions.

Solitary genital skin lesions and lesions involving the palms and soles may easily lead to misdiagnosis as syphilis and other STIs, which may, in turn, delay detection [47]. To date, the current spread has disproportionately affected men who are gay or bisexual and other men who have sex with men (MSM), which suggests amplification of transmission through sexual networks. Sexual activity, largely among gay or bisexual men, was by far the most frequently suspected route of transmission. The strong likelihood of sexual transmission was supported by the findings of primary genital, anal, and oral mucosal lesions, which may represent the inoculation site [47], as depicted in Figure 2. MPXV deoxyribonucleic acid (DNA) was detected in seminal fluid, supporting this hypothesis. However, whether semen is capable of transmitting infection remains to be investigated [47]. Reports of clusters associated with sex parties or saunas further underscore the potential role of sexual contact as a promoter of transmission. International travel and attendance at large gatherings linked to sex-on-site activities may explain the global spread of MPXV infections amplified through sexual networks [48]. The likelihood of sustained community transmission cannot be ruled out, and the extent to which pre-symptomatic or asymptomatic infection may occur as the infectious period is unknown, as well as the further spread of MPXV among persons with multiple sexual partners in interconnected networks and the likely role of mass gatherings [44].

The clinical presentation of monkeypox cases associated with this outbreak has been atypical as compared to previously documented reports: many cases in newly affected areas are not presenting with the classically described clinical picture for monkeypox (fever, swollen lymph nodes, followed by a centrifugal rash) (Table 7) [44].

There is no treatment specifically for human monkeypox. However, monkeypox and smallpox viruses are genetically similar, which means that vaccines and antiviral drugs developed to protect against smallpox may be used to prevent and treat MPXV infections. A study in 1988 found that the smallpox vaccine was around 85% protective in preventing MPXV infection in close contacts and in lessening the severity of the disease [48]. A newer smallpox and monkeypox vaccine based on modified vaccinia Ankara has been approved, but with limited availability [14]. Other measures of prevention include regular hand washing and avoiding sick people and animals [49]. Antiviral drugs, cidofovir and tecovirimat, vaccine immune globulin, and the smallpox vaccine may be used during outbreaks [42,50]. The illness is usually mild, and most of those infected will recover within a few weeks without treatment [42].

Various wild mammals have been identified as susceptible to MPXV in areas that have previously reported monkeypox. Thus far, in this new outbreak, there is no documented evidence of domestic animals or livestock being affected by MPXV, except for one case of a dog with confirmed MPXV infection that might have been acquired through human transmission [51]. This suggests the need to keep pets isolated from human-infected patients, and secondary transmissions via pets require further investigation (Table 8).

## 4. Conclusions

Monkeypox is primarily endemic to remote regions of the rainforest of West and Central Africa, where it has been mainly transmitted from the animal reservoir(s) to humans. Since May 2022, this viral disease of zoonotic origin represents the most recent PHEIC, when thousands of cases of human monkeypox have been identified in several non-endemic countries, mainly in Europe and North America, emerging in separated populations without apparent link between the clusters, marked by human-to-human transmission, and caused by people’s movement at a global level. Although the current worldwide outbreak is disproportionately affecting MSM, the spread to other populations has been anticipated, thus suggesting changes both in biological aspects of the virus and in human behavior. Such changes might be driven by waning smallpox immunity, resumption of international travel, following the relaxation of coronavirus (COVID-19) prevention measures, and sexual interactions associated with large gatherings.

## Figures and Tables

**Table 1:** Relevant developments in the history of monkeypox (1958–2022).

Year	Developments
1958	Monkeypox virus discovered in captive *Macaca fascicularis* monkeys
1970	First recognized case of human monkeypox in a 9-month-old child in DRC
1970–1979	Forty-seven human monkey cases reported in West and Central Africa, 81% of them in DRC
1981–1986	WHO monitoring project of human monkeypox in DRC
1996	Sudden increase in the number of human monkeypox cases reported in DRC, after a period of 10 years of persistent decrease in the number of cases
2003	First outbreak of human monkeypox outside its endemic region, registered in the USA, and indirectly related to various rodent species infected imported from West Africa
2017	The largest outbreak of human monkeypox on record in West Africa, ongoing in Nigeria
2018–2019	Linked with the Nigeria 2017 outbreak, imported cases of human monkeypox were registered in the United Kingdom, Israel, and Singapore
2022	Human monkeypox outbreaks occur in several countries outside endemic areas, mainly in Europe and North America. WHO determined this new outbreak a Public Health Emergency of International Concerns. First report of transmission of monkeypox virus from an infected human to an animal (a companion dog)

DRC, Democratic Republic of Congo (ex-Zaire); USA, United States of America; WHO, World Health Organization.

**Table 2:** Identified animals which can be infected with Monkeypox virus (adapted from [7]).

Rodents	Carnivores	Insectivores	Non-Human Primates
Prairie dog	Dog	Hedgehog Shrew	Monkey Ape
Squirrel			
Marmot			
Groundhog			
Chinchilla			
Giant-pouched rat			

**Table 3:** Former and new classification of monkeypox virus clades, geographic distribution, clinical outcomes, and case-fatality rates (adapted from [9,10,11,12,13]).

Former Classification	New Classification	Geographic Distribution	Clinical Outcomes	Case Fatality Rate
Congo Basin clade	Clade I	Central African and Western African outbreaks and localized spillover events in global north countries from both human and non-human host	More clinically severe, with higher mortality rates and exhibited increased transmissibility	>10%
Western African clade	Clade IIa		Associated with milder infection, lower mortality rates, and reduced transmissibility	<1%
	Clade IIb	2017–2019 outbreaks from the UK, Israel, Nigeria, USA, and Singapore, and 2022 global outbreak from a human host	Ongoing outbreak currently under investigation	

UK, United Kingdom; USA, United States of America.

**Table 4:** Comparison of monkeypox clinical presentation and death rates with smallpox and chickenpox (adapted from [14]).

Characteristics	Monkeypox	Smallpox	Chickenpox
Virus	Monkeypox virus	Variola virus	Varicella-zoster virus
Fever	1–3 days before rash	1–3 days before rash	1–2 days before rash
Rash	Rash often in one stage of development; slow development; lesions denser on the face, and present on palms and soles	Rash often in one stage of development; slow development; lesions denser on the face and in mucous membranes of the nose and mouth, and present on palms and soles	Rash often in multiple stages of development; rapid development; lesions denser on the trunk and absent on palms and soles
Lymphadenopathy	Present	Absent	Absent
Death	Up to 10%	Up to 30%	Rare

**Table 5:** Diagnostic tests overview for MPXV, including disease stage and suitable samples to be collected.

Tests	Disease Stage	Suitable Samples	Description
NAAT (PCR)	Febrile stage	Oropharyngeal swab or/and EDTA blood	Detects DNA MPXV and identifies the viral clade. NAAT can be generic to OPXV or specific to MPXV
	Rash stage	Skin lesion material ^a^	
Immunohistochemistry (antigen detection)	Rash stage	Skin lesion material	Uses an antibody directed against an OPXV antigen and proves the presence of a current infection. Not specific for MPXV
Viral culture/isolation	Rash stage	Skin lesion material	Detects viral particles. Requires facilities, appropriate expertise, and skills. Time and resource intensive. Not specific for MPXV
Electron microscopy	Rash stage	Skin lesion material or viral culture	Identify virus morphology. Requires facilities, appropriate expertise, and skills. Time and resource intensive. Not specific for MPXV
Serological analysis for specific antibodies (IgM and IgG antibody detection)	Rash stage	Serum or plasma (two samples, at 21 days apart, first sample collected during the first week of illness)	Uses an antigen directed against the antibody but has a limited diagnostic value because it proves a current or past infection/vaccination. Not specific for MPXV
	Recovery		

^a^ Other clinical samples can be considered for research purposes. DNA, Deoxyribonucleic acid; EDTA, Ethylenediamine tetraacetic acid; IgG, immunoglobulin G; IgM, immunoglobulin M; MPXV, Monkeypox virus; NAAT, Nucleic Acid Amplification Testing; OPXV, Orthopoxvirus; PCR, Polymerase Chain Reaction.

**Table 6:** Top 20 countries with reported monkeypox cases of the 2022 current outbreak until September 2022 (adapted from [43]).

Country	Cases	Country	Cases
United States of America	24,363	Netherlands	1221
Spain	7083	Portugal	908
Brazil	7019	Italy	837
France	3898	Belgium	757
Germany	3570	Chile	728
United Kingdom	3552	Switzerland	502
Peru	2091	Austria	304
Colombia	1653	Nigeria	277
Mexico	1367	Argentina	265
Canada	1363	Israel	250

**Table 7:** Atypical features of the new monkeypox outbreak (adapted from [44]).

▪ Few lesions ▪ No lesions, but anal pain and bleeding ▪ Genital or perineal/perianal alone lesions ▪ Lesions at different stages of development ▪ Absence of prodromal period or constitutional symptoms appearing after the lesions

**Table 8:** Measures to avoid human-to-animal and animal-to-human transmission of monkeypox virus (Adapted from [44]).

▪ Avoid close contact between monkeypox-infected people and domestic pets ▪ Proper waste management to prevent the transmission from infected humans to pets, peri-domestic animals, animals in zoos, and animals from the wild ▪ Those leaving or traveling to endemic areas of monkeypox should avoid contact with potential reservoirs of monkeypox virus or eating bush meat

## Data Availability

Not applicable.
